# Role of Adiponectin in Central Nervous System Disorders

**DOI:** 10.1155/2018/4593530

**Published:** 2018-07-29

**Authors:** Jenna Bloemer, Priyanka D. Pinky, Manoj Govindarajulu, Hao Hong, Robert Judd, Rajesh H. Amin, Timothy Moore, Muralikrishnan Dhanasekaran, Miranda N. Reed, Vishnu Suppiramaniam

**Affiliations:** ^1^Department of Drug Discovery and Development, Auburn University, Auburn, AL, USA; ^2^Department of Pharmacology, Key Laboratory of Neuropsychiatric Diseases, China Pharmaceutical University, Nanjing, China; ^3^Department of Anatomy, Physiology and Pharmacology, College of Veterinary Medicine, Auburn University, Auburn, AL, USA; ^4^Center for Neuroscience, Auburn University, Auburn, AL, USA

## Abstract

Adiponectin, the most abundant plasma adipokine, plays an important role in the regulation of glucose and lipid metabolism. Adiponectin also possesses insulin-sensitizing, anti-inflammatory, angiogenic, and vasodilatory properties which may influence central nervous system (CNS) disorders. Although initially not thought to cross the blood-brain barrier, adiponectin enters the brain through peripheral circulation. In the brain, adiponectin signaling through its receptors, AdipoR1 and AdipoR2, directly influences important brain functions such as energy homeostasis, hippocampal neurogenesis, and synaptic plasticity. Overall, based on its central and peripheral actions, recent evidence indicates that adiponectin has neuroprotective, antiatherogenic, and antidepressant effects. However, these findings are not without controversy as human observational studies report differing correlations between plasma adiponectin levels and incidence of CNS disorders. Despite these controversies, adiponectin is gaining attention as a potential therapeutic target for diverse CNS disorders, such as stroke, Alzheimer's disease, anxiety, and depression. Evidence regarding the emerging role for adiponectin in these disorders is discussed in the current review.

## 1. Introduction

Adiponectin, a hormone produced by adipocytes, regulates metabolic processes and improves insulin sensitivity. Adiponectin signaling has been widely studied in multisystem diseases, for example, obesity, diabetes, dyslipidemia, atherosclerosis, and comorbid metabolic dysfunction in the setting of cardiovascular disease such as hypertension. Serum adiponectin levels appear to be inversely correlated with the presence and severity of metabolic dysfunction, that is, lower serum adiponectin is seen in patients with disease [1–5]. Serum adiponectin levels also appear to be altered in various neurological disorders in which the etiologies of these conditions involve both metabolic and inflammatory components. Furthermore, adiponectin receptors are highly expressed in a number of brain regions, and adiponectin exerts neuroprotective and antidepressant properties, likely through specific adiponectin receptors expressed in the central nervous system (CNS). Therefore, this review provides an overview of adiponectin and discusses recent evidence supporting adiponectin's role in stroke, Alzheimer's disease (AD), anxiety, and depression.

## 2. Overview of Adiponectin

### 2.1. Structure and Production of Adiponectin

Adiponectin is a 244-amino acid polypeptide protein which belongs to the complement 1q family [6]. Adiponectin is produced in adipocytes, and its transcription is regulated by sirtuin 1/forkhead box O-1 and peroxisome proliferator-activated receptors (PPARs) [7]. It forms a characteristic homomultimer composed of an NH_2_-terminal collagenous region and a COOH-terminal globular domain [8]. Usually, adiponectin exists as a full-length protein of 30 kDa (fAd) that circulates in trimeric, hexameric, and higher order complexes [9]. Adiponectin is further identified based on the molecular weight of these multimers as low (trimer), middle (hexameric), and high (higher order complexes) [10]. In the circulation, adiponectin is present as either these full-length forms or a smaller, globular fragment. Full-length adiponectin is cleaved by leukocyte esterase to form globular adiponectin (gAd). Both gAd and fAd mediate tissue-specific effects, as well as regulate distinct signaling pathways in the same tissue. Studies report that a sexual dimorphism exists in serum adiponectin levels. Adiponectin serum levels are approximately 2.5-fold higher in female than in male mice [11], and this sexual dimorphism is also confirmed in humans [12]. Furthermore, the associations between adiponectin and certain disease states appear to be sex specific [13–15]. Because adiponectin may have sex-specific effects, differentiating the effects of altered adiponectin levels in both males and females is important, although the vast majority of rodent studies have only been performed in males.

It was initially thought that adiponectin does not cross the blood-brain barrier (BBB) [16]. However, adiponectin is observed in human cerebral spinal fluid (CSF) [17, 18], with evidence that the adiponectin trimer is the predominate form [17]. In addition, studies in mice show that peripheral intravenous application of adiponectin leads to a concurrent rise in CSF adiponectin [19]. Therefore, adiponectin does cross the BBB, although concentrations in the CSF are approximately 1000-fold lower than that in serum [17].

### 2.2. Adiponectin Receptors

Adiponectin is known to bind 3 receptors: adiponectin receptor 1 (AdipoR1), adiponectin receptor 2 (AdipoR2), and T-cadherin. AdipoR1 and AdipoR2 were isolated from a human skeletal muscle cDNA library with AdipoR2 showing >60% homology to AdipoR1 [20]. Though AdipoR1 and AdipoR2 are surface membrane proteins containing seven transmembrane domains, they differ from other G protein-coupled receptors, because the amino terminal of the receptor is located intracellularly while the carboxyl terminal is located extracellularly [21]. AdipoR1 and AdipoR2 are expressed abundantly in the liver, muscle, brain, and adipose tissue in humans. These receptors have differing affinities for specific forms of adiponectin. While AdipoR1 is a high-affinity receptor for gAd, it acts as a low-affinity receptor for fAd in skeletal muscle. In contrast, AdipoR2 is an intermediate-affinity receptor for both gAd and fAd in the liver [22]. Mouse studies indicate that AdipoR1 and AdipoR2 mediate metabolic actions of adiponectin in peripheral tissues [23]. These effects are apparent in AdipoR1-AdipoR2 double knockout mice, which are glucose intolerant and hyperinsulinemic under certain conditions. This indicates that AdipoR1 and AdipoR2 are important for regulating basal glucose levels and insulin sensitivity. In the CNS, AdipoR1 and AdipoR2 are expressed in various areas of the brain, including the hypothalamus, brainstem, hippocampus, and cortex [24]. In the hypothalamus and brainstem, adiponectin is thought to regulate food intake and energy expenditure via AdipoR1-mediated AMP-activated protein kinase (AMPK) signaling [25]. This signaling may relate to the association between adiponectin and metabolic disease including atherosclerosis, which is a precipitating factor for stroke. In the hippocampus, adiponectin appears to promote neurogenesis via AdipoR1 [26] and directly affects synaptic function via AdipoR2 [27]. Adiponectin signaling in various areas of the brain, including the cortex, appears to be neuroprotective against damage induced by metabolic insults, such as a high-fat diet, in part through AdipoR1 [28].

High molecular weight forms of adiponectin can bind to T-cadherin, which is traditionally thought of as a receptor that binds low-density lipoprotein (LDL) with signaling cascades related to cell growth, proliferation, and migration. T-cadherin, a glycosylphosphatidylinositol-anchored membrane protein, is abundantly present in the cardiovascular system [29]. Deficiency in T-cadherin leads to exacerbation of cardiac hypertrophy during chronic pressure overload, suggesting a cardioprotective role of T-cadherin [30]. T-cadherin is a unique receptor, because it lacks cytoplasmic and transmembrane domains [30]. Therefore, the mechanism by which T-cadherin influences intracellular signaling is unclear, and it has been suggested that this receptor may require interaction with transmembrane proteins for some physiological actions [31].

Several studies have established a link between the gene that encodes T-cadherin, cadherin 13, and various metabolic diseases [32–34]. Furthermore, in human aortic smooth muscle cells, adiponectin's ability to inhibit inflammation was negated after knockdown of T-cadherin [35]. This evidence suggests that adiponectin interactions with T-cadherin may account for some of adiponectin's metabolic effects. T-cadherin is also present in the brain [36]. However, whether interactions between adiponectin and T-cadherin occur in the brain is unclear. T-cadherin's preference for binding high molecular weight adiponectin isomers over lower molecular weight isoforms [29] may preclude the interaction, as the major adiponectin isoform in the brain is the trimeric form (~80%) with the other 20% predominately being lower molecular weight forms [17]. Because metabolic dysfunction may be a precipitating factor in cognitive dysfunction [37, 38], the mechanistic relationship between peripheral adiponectin signaling through T-cadherin and any downstream effects on cognition should be further explored.

### 2.3. Peripheral Adiponectin Receptor Signaling

Adiponectin receptor signaling in the periphery regulates insulin sensitivity, reduces oxidative stress, and inhibits inflammation. In the downstream signaling pathways of AdipoR1 and AdipoR2, the adapter protein containing a pleckstrin homology domain, phosphotyrosine binding domain, and leucine zipper motif (APPL1) links the adiponectin receptors to various signaling molecules including AMPK and p38 mitogen-activated protein kinase (p38MAPK), which are important for many metabolic actions of adiponectin [39]. AMPK further mediates important downstream effects of adiponectin, including enhanced insulin sensitivity in part through serine phosphorylation of insulin receptor substrate 1 (IRS-1) [40]. While AdipoR1 regulates insulin sensitivity via activation of the AMPK pathway, AdipoR2 is more involved with activation of the peroxisome proliferator-activated receptor alpha (PPAR*α*) pathway, which stimulates energy dissipation and inhibits inflammation and oxidative stress [23].

Overall, adiponectin appears to reduce inflammation cascades *in vivo* and *in vitro* by several mechanisms, although there is some conflicting data concerning overall effects in immunological diseases [41]. Adiponectin decreases the expression of proinflammatory cytokines such as tumor necrosis factor-*α* (TNF-*α*) [42] and increases the expression of anti-inflammatory molecules such as interleukin 10 (IL-10) [43]. In return, some proinflammatory factors, such as TNF-*α* and IL-6, appear to inhibit the production of adiponectin [44] suggesting bidirectional modulation.

Adiponectin also has a role in the reduction of oxidative stress through AMPK-mediated reduction of reactive oxygen species [45]. Furthermore, disruption of AdipoR1 and AdipoR2 signaling results in significantly increased expression of genes encoding chemokines, such as chemokine C-C motif ligand 2, and decreased expression of genes encoding molecules that reduce oxidative stress [23]. In pancreatic beta cells and cardiomyocytes, overexpression of adiponectin decreases caspase-8-mediated cell death, whereas genetic ablation of adiponectin enhances apoptosis [46, 47]. In summary, the proposed beneficial effects of adiponectin in the periphery include improved insulin sensitivity, reduced inflammation, and reduced oxidative stress.

While the previously mentioned peripheral effects of adiponectin are mainly due to AdipoR1 and AdipoR2 signaling, less is known about adiponectin-T-cadherin-specific signaling. Overall, T-cadherin appears to be important in antiatherogenesis and cardioprotection. T-cadherin is necessary for adiponectin-mediated phosphorylation of AMPK in some models of ischemia [48]. Interestingly, T-cadherin may not independently mediate signaling, but instead may function to localize circulating adiponectin at target tissues. Following ischemia, T-cadherin appears to localize adiponectin to vascular tissue, where adiponectin promotes revascularization and prevents atherosclerotic plaque formation [31, 35].

### 2.4. Central Adiponectin Receptor Signaling

In the brain, adiponectin appears to play a role not only in energy homeostasis, but also in neuroprotection in various disease states. In the hypothalamus, adiponectin signaling influences satiety, as well as energy homeostasis. Intracerebroventricular (i.c.v.) delivery of adiponectin improves peripheral insulin sensitivity and glucose homeostasis [49], suggesting that central actions of adiponectin may also influence metabolic diseases. In the hippocampus, adiponectin signaling regulates neurogenesis and synaptic plasticity. *In vitro*, adiponectin increases proliferation in hippocampal progenitor cells and Neuro2a cells through AdipoR1 signaling [26]. In the hippocampal dentate gyrus (DG) of adult male mice, reductions in adiponectin lead to reduced neurogenesis, and adiponectin i.c.v. infusion increases neurogenesis [50]. This effect is mediated by activation of p38MAPK and the resultant inactivation of glycogen synthase kinase 3 beta via phosphorylation of Ser-389 [51]. A reduction in adult neurogenesis may be linked to depression since stressful conditions reduce hippocampal neurogenesis, whereas antidepressant treatment increases neurogenesis [52]. However, a causal relationship for reduced neurogenesis in depression is not well established [53]. Thus, additional research is required to elucidate a possible role for promotion of neurogenesis via adiponectin as a therapeutic option in treatment of depression.

### 2.5. Summary

**Figure 1:**
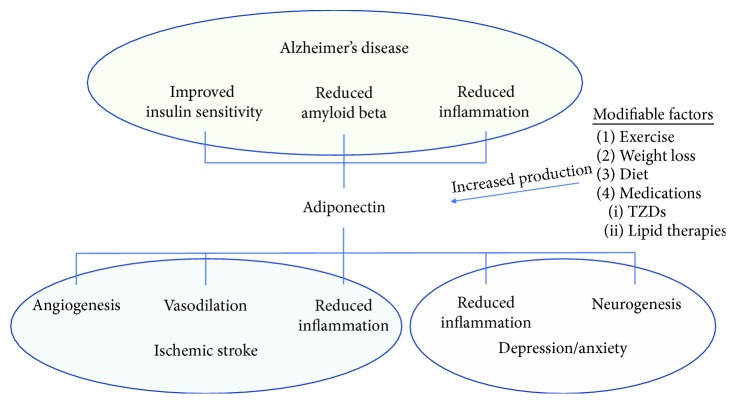
Proposed beneficial effects of adiponectin in central nervous system disorders. Adiponectin receptor signaling is under investigation for central nervous system disorders including Alzheimer's disease (AD), ischemic stroke, and depression. In models of AD, adiponectin receptor activation appears to reduce amyloid beta and improve insulin sensitivity. Adiponectin receptor signaling increases angiogenesis and enhances vasodilation, which may be of benefit in the treatment or prevention of ischemic stroke. Reductions in inflammation related to adiponectin may be beneficial to decrease risk of various diseases, including ischemic stroke, AD, and depression/anxiety. Adult neurogenesis may be reduced in depression, and adiponectin promotes neurogenesis especially in response to exercise. There are a number of modifiable factors that enhance production of adiponectin and may reduce risk of the previously mentioned disorders. In particular, exercise, weight loss, a diet rich in omega-3 polyunsaturated fatty acids, and certain medications such as lipid-lowering therapies and thiazolidinediones (TZDs) all increase circulating adiponectin levels.

Our understanding of adiponectin and its role in maintaining metabolic homeostasis and modulating various systemic disease states has greatly increased over the past decade. The role for adiponectin in CNS disorders is becoming more appreciated, although much remains unknown. The remainder of this review will focus on the role of adiponectin in cerebrovascular disease/stroke, mild cognitive impairment (MCI) and AD, anxiety, and depression (Figure 1). A summary of major studies related to the neuroprotective effects of adiponectin in these CNS disorders is provided in Table 1.

## 3. Adiponectin in Atherosclerosis/Stroke

### 3.1. Overview and Adiponectin Levels in Stroke

According to American Heart Association Statistics Committee and Stroke Statistics Subcommittee, approximately 700,000 people experience a new or recurrent stroke every year, and on an average, every 45 seconds someone in the United States suffers from a stroke [54]. Since adiponectin is associated with many cardiovascular risk factors, such as hypertension, type II diabetes, and an altered lipid profile, a link between adiponectin and stroke is expected [55]. However, a meta-analysis of prospective cohort studies determined that plasma adiponectin levels are not a risk factor for the occurrence of the disease [56]. Yet, low plasma adiponectin levels have been observed poststroke, and in fact, decreased adiponectin levels predict increased risk of 5-year mortality after a first-ever ischemic stroke [57]. Thus, the utility of plasma adiponectin levels in management of risk factors for stroke versus management of poststroke sequelae may differ, and this difference may be due to varying mechanisms of action elicited by adiponectin receptor signaling under differing conditions.

### 3.2. Adiponectin Protective Effects in Stroke

Despite unclear data on the correlations between serum adiponectin levels and stroke risk or recovery from stroke, there are several studies demonstrating adiponectin-mediated mechanistic effects that are protective against atherosclerosis as well as stroke pathogenesis. Circulating adiponectin inhibits monocyte adhesion to endothelial cells [58] and inhibits macrophage transformation to foam cells by reducing oxidized LDL binding and uptake [59]. This process is a crucial step in atherosclerosis as well as stroke pathogenesis. Adiponectin also inhibits induction of vascular cell adhesion molecule-1 (VCAM-1) and intracellular cell adhesion molecule-1 (ICAM-1) [60], which typically bind to leukocytes and initiate formation of atheroma following endothelial cell injury [61]. The mechanism for reduction in adhesion molecules may be through activation of AdipoR2 which increases PPAR*α* activity [23], since PPAR*α* agonists can also reduce VCAM-1 [62] and ICAM-1 [63]. Interestingly, a clinical trial with the PPAR*γ* agonist rosiglitazone resulted in increased circulating levels of adiponectin and reduced circulating VCAM-1 [64], indicating that PPAR*γ* agonists may confer antiatherogenic properties [65] through an increase in adiponectin.

Other studies show that elevated plasma adiponectin protects endothelial cells from hypercholesterolemia-induced vascular injury and suppresses the uptake of modified LDL into foam cells. In mouse models of atherosclerosis, adiponectin reduces the size of atherosclerotic lesions [66], whereas adiponectin knockout (APN-KO) mice exhibit excessive vascular remodeling in response to acute ischemic insult [67]. While APN-KO mice exhibit increases in breadth of cerebral infarct after ischemia-reperfusion, exogenous adiponectin reduces the infarct size in both APN-KO and wild type mice. Furthermore, adiponectin overexpression increases indices of positive behavioral outcomes as well as stimulates angiogenesis following ischemic injury [68]. Therefore, adiponectin may regulate vascular remodeling, confer antiatherogenic properties within the vasculature, and afford protection against stroke and/or stroke severity.

### 3.3. Adiponectin Influences Nitric Oxide

Protective actions of adiponectin in stroke may also be due to stimulation of nitric oxide (NO) synthesis from endothelial cells [69], through AdipoR1 signaling [70, 71]. In case of acute stroke, intense vasospasm may occur over the first few weeks which can lead to increased morbidity and mortality. NO plays a significant protective role, since it causes vasodilation and increases blood flow [72]. Adiponectin increases NO production through stimulation endothelial NO synthase (eNOS), and therefore, adiponectin may be neuroprotective following stroke [70]. Usually, plasma NO production is increased in response to hypoxia/ischemia, but this response is absent in APN-KO mice [69]. Adiponectin treatment of bovine aortic endothelial cells increases NO production significantly, an effect mediated by phosphorylation of both Akt at Ser^473^ and eNOS at Ser^1179^ via phosphatidylinositol-4,5-bisphosphate 3-kinase (PI3K) [73]. Akt can also directly phosphorylate eNOS at Ser^117^, resulting in NO production and the regulation of vasomotor responses [74]. Phosphorylation of Akt is activated by calmodulin-dependent protein kinase kinase (CaMKK), which then concomitantly phosphorylates eNOS in response to increased calcium in the cytoplasm [75]. The calcium-dependent activation of calmodulin stimulates CaMKK and the recruitment of calmodulin to eNOS, causing a “burst-like” release of NO. AMPK, which is also activated by adiponectin signaling, activates cardiac and endothelial cell eNOS by phosphorylation at Ser^1177^ (human sequence) *in vitro* [56]. AMPK signaling is also required for vascular endothelial growth factor -stimulated endothelial cell NO production, migration, and differentiation in response to hypoxic conditions and increased oxidative stress in stroke [76], which also promotes angiogenesis in vivo. Therefore, adiponectin may play a significant protective role in stroke via influence on NO.

### 3.4. Summary

**Figure 2:**
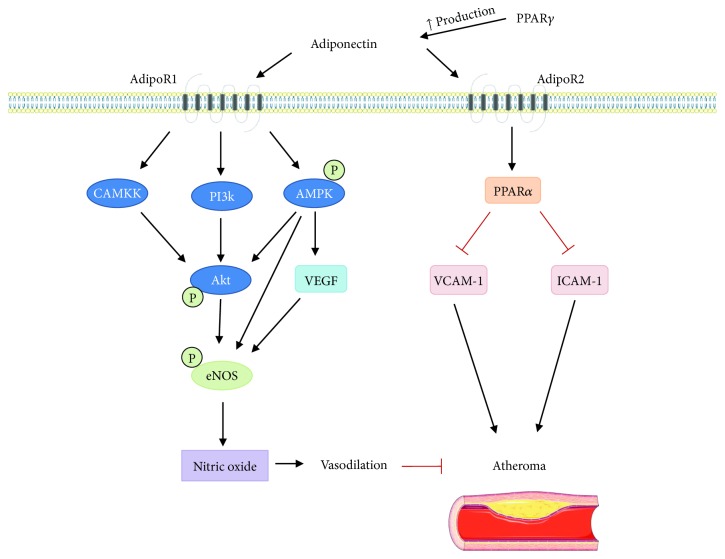
Proposed signaling mechanisms of adiponectin in prevention of ischemic stroke. Signaling through AdipoR1 and AdipoR2 can reduce formation of atheroma. AdipoR1 activates the AMP-activated protein kinase (AMPK) pathway resulting in phosphorylation of protein kinase B (Akt) and activation of vascular endothelial growth factor (VEGF). Activation of Akt through calcium calmodulin kinase kinase (CAMKK), phosphatidylinositol-4,5-bisphosphate 3-kinase (PI3K), and AMPK contributes to activation of endothelial nitric oxide synthase (eNOS). Additionally, AMPK and VEGF also increase eNOS activity leading to nitric oxide (NO) production. Increase in production of NO leads to vasodilation, which is beneficial in prevention of atheroma and ischemia. Adiponectin signaling reduces vascular cell adhesion molecule 1 (VCAM-1) and intracellular adhesion molecule 1 (ICAM-1), and these adhesion molecules increase atheroma size. Peroxisome proliferator-activated receptor alpha (PPAR*α*) also reduces VCAM-1 and ICAM-1, and PPAR*α* is activated by AdipoR2 signaling. PPAR*γ* increases production of adiponectin and also leads to reduction of VCAM-1 and ICAM-1. This figure was produced using Servier Medical Art (http://www.servier.com/).

Because adiponectin reduces cerebrovascular infarct size and improves behavioral outcomes following cerebral ischemia, therapeutic strategies targeting the adiponectin signaling pathways (Figure 2) should be considered for further study. However, achieving beneficial therapeutic concentrations of adiponectin has been difficult due to its high serum concentrations and multimeric structure. Therefore, research designed to develop novel adiponectin mimetics and/or receptor agonists which mimic physiological functions of adiponectin are currently under development.

## 4. Adiponectin and Alzheimer's Disease

### 4.1. Overview and Adiponectin Levels in MCI and AD

Currently, more than 5 million Americans are living with AD, and death rates from AD have increased by 89% since 2000 [77]. AD is associated with brain insulin resistance, leading to the possibility that the insulin-sensitizing properties of adiponectin may confer protection in AD and other cognitive disorders [78–80]. Similar to the studies on correlations between adiponectin levels and stroke risk or stroke recovery, contrasting reports exist on the association between adiponectin levels and MCI and/or AD. In a cohort of participants from the Framingham Heart Study, elevated serum adiponectin levels were associated with increased risk for development of AD and dementia in women, but not in men [81]. Additional studies of serum adiponectin levels in MCI and AD have found an increase [82–84], decrease [85], or no change [86, 87] in adiponectin levels. Several factors may account for these disparate findings. For example, one strong predictor of circulating adiponectin levels is body weight; increased body weight is inversely correlated to adiponectin levels [88]. In MCI and AD patients, weight is reduced, [89] which may explain the increase in serum adiponectin in some studies. However, when the Framingham Heart Study adjusted for age, body mass index (BMI), and weight change, there was still a significant increase in the risk of AD, but not all-cause dementia, in women with elevated adiponectin levels [81]. Diet is another consideration for adiponectin levels, as increased intake of omega-3 polyunsaturated fatty acids increases plasma adiponectin [90]. Another potential confounder is drug therapies that affect adiponectin levels [91]. Drugs used in AD treatment, such as acetylcholinesterase inhibitors, may increase levels of adiponectin [92]. Of note, most studies examining serum levels of adiponectin in MCI/AD have not adjusted for medication factors. One group reported increased serum adiponectin levels following treatment with donepezil, but BMI was concurrently reduced, which confounds the results [92]. Thiazolidinediones [93, 94], which are PPAR*γ* agonists, and lipid-lowering drugs such as niacin [95, 96], fibrates [97, 98], and certain statins [99–101] can also increase adiponectin levels.

Studies are now also evaluating correlations between CSF adiponectin levels and disease onset or progression. In one study, a reduction in CSF adiponectin in AD patients was reported with a concurrent increase in serum adiponectin [102]. In the CSF of these patients, adiponectin was negatively correlated with hyperphosphorylated tau (p-tau), but in contrast, adiponectin was positively correlated with amyloid beta (A*β*). This is interesting as p-tau containing tangles and A*β* plaques are both hallmarks of AD. Reduced adiponectin CSF levels were correlated with declining scores on the mini-mental status exam, a clinical test for cognitive function in which higher scores indicate better memory. This may indicate that as AD progresses, adiponectin in the CSF decreases. Adiponectin was also colocalized with p-tau in neurofibrillary tangles in AD, suggesting that sequestering may occur, which could explain the reduced CSF adiponectin levels [102]. In a separate study, adiponectin levels were increased in the CSF of patients with MCI, but this correlation was not present in AD [82]. Based on these studies, one hypothesis is that increased adiponectin in the CSF may initially indicate MCI, but declining levels may mark disease progression (from MCI to AD). It remains difficult to make conclusions from correlative data on adiponectin levels alone without mechanistic studies, given that adiponectin levels are affected by multiple intrinsic and extrinsic factors.

### 4.2. Reduced Adiponectin Signaling and Cognitive Deficits

There is growing interest in the role of adiponectin in memory and synaptic plasticity. APN-KO mice historically used to examine peripheral metabolic disorders are now being used to investigate the role of adiponectin in the brain. In one study, APN-KO mice were utilized as a model of insulin resistance and associated memory pathology [103]. The APN-KO mice displayed a number of behavioral changes indicating memory impairments and increased anxiety at 9 and 18 months. Furthermore, the APN-KO mice had increased protein levels of A*β*_42_ and p-tau, pathological markers of AD, in various areas of the brain by 18 months of age compared to age-matched controls. These knockout mice also displayed cerebral insulin resistance and reduced AMPK activation, which is a potential mechanism for the cognitive deficits and pathological markers observed.

A separate group utilized AdipoR1 knockdown mice via shRNA tail vein injection [104]. These mice exhibited approximately a 50% reduction in AdipoR1 protein expression in the brain, liver, kidney, and spleen with metabolic dysfunctions including increased body weight, increased LDL, and hepatic dysfunction after about 6 weeks of shRNA injections. Interestingly, these animals displayed a similar phenotype to APN-KO mice in terms of central effects, including deficits in hippocampal-dependent Morris water maze performance, hippocampal neuronal loss, and increased A*β*. In fact, these deficits were seen in 11-week-old mice after only about 5-6 weeks of AdipoR1 knockdown. Therefore, it appears that reduction of adiponectin or AdipoR1 signaling negatively affects cognition and leads to increases in pathogenic markers of AD. The role of AdipoR2 is less clear. It is noted that in the periphery, both AdipoR1 signaling and AdipoR2 signaling appear to be beneficial in reducing metabolic dysfunction [105], suggesting that signaling through AdipoR2 may also affect cognition. It is also unclear whether the central effects noted in knockout or knockdown mice are the result of peripheral changes, central changes, or a combination of both. Additional mechanistic studies will be highly useful to determine the role of central versus peripheral adiponectin.

### 4.3. Adiponectin and Brain Insulin Signaling

As reduced insulin signaling is associated with cognitive impairment and AD [106], adiponectin may influence cognition via reductions in insulin resistance. In human studies, AD patients appear to have reduced insulin activity in the brain as demonstrated by elevated levels of phosphorylated IRS-1 at Ser^616^ and Ser^636^ [79] along with reduced insulin receptor (IR) expression [107]. Based on this, there is an immense interest in the use of insulin-sensitizing agents in animal models of AD [108–110], and there is a phase II/III clinical trial examining intranasal insulin in MCI or mild AD with results from the trial expected to emerge soon. As adiponectin is an insulin-sensitizing hormone, there is interest in adiponectin as a treatment for AD to restore insulin signaling in the brain. As expected, aged APN-KO mice have increased pIRS-1 at Ser^616^ and reduced IR levels in the frontal cortex indicating brain insulin resistance [103]. However, unexpectedly, these aged APN-KO mice have increased IR expression in the hippocampus. This may be due to compensatory mechanisms, and despite the increase in IR, the aged APN-KO animals still displayed neuroinflammation, increased apoptosis, and synaptic loss in the hippocampus. In the human neuroblastoma SH-SY5Y cell line, adiponectin enhances insulin sensitivity via activation of AMPK through AdipoR1 signaling [103]. Therefore, adiponectin receptor signaling and effects on insulin sensitivity in the brain may be an interesting mechanism to target in the treatment of AD.

### 4.4. Adiponectin Acute Effects at Hippocampal Synapses

Adiponectin may also influence cognition by directly affecting hippocampal synaptic plasticity. Adiponectin injection in the DG of the hippocampus leads to a chemical long-term potentiation (LTP) characterized by a 20% increase in excitatory post-synaptic potential slope [111], indicating that adiponectin may have a beneficial effect on memory. Furthermore, adiponectin applied 10 minutes prior to stimulation protocols potentiates LTP via high-frequency stimulation and prevents induction of long-term depression (LTD) via low-frequency stimulation, suggesting that adiponectin may play a role in both LTP and LTD. In contrast to the DG, in the cornu ammonis 1 (CA1) region of the hippocampus, the adiponectin receptor agonist AdipoRon reduces LTP [112], suggesting subregional variation in adiponectin responses. In addition, these studies found subregion-specific alterations in paired pulse facilitation, indicating potential presynaptic mechanisms of adiponectin signaling. As both AdipoR1 and AdipoR2 are expressed in the hippocampus, it is unclear which receptor is responsible for the synaptic plasticity effects in these regions of the hippocampus. Interestingly, AdipoR2 deletion, but not AdipoR1 deletion, leads to increased hyperexcitability in DG neurons in acute brain slices [27]. Thus, it appears that adiponectin signaling has direct and acute effects on hippocampal synapses, and further research in this area is needed.

### 4.5. Neuroprotective Properties of Adiponectin

Despite some controversy in human correlational studies, there is strong evidence for the neuroprotective effects of adiponectin in cell culture and animal models [113]. Many studies in cell culture models have described neuroprotective properties of adiponectin against neurotoxic insults. Adiponectin is protective against kainate-induced excitotoxicity [114], A*β* toxicity during oxidative stress [115], and high glucose concentrations [28]. Many of these effects appear to be due to increased AMPK signaling [114, 115]. Interestingly, adiponectin signaling also can occur in astrocytes where it may actually promote expression of proinflammatory cytokines [116], but relatively little work has been done to delineate the role of adiponectin signaling in these cells. In the periphery, adiponectin promotes anti-inflammatory mediators and inhibits production of TNF*α* [117]. Therefore, it will be interesting to determine how adiponectin may differentially influence inflammation in the periphery versus the CNS and the role of glia cells in adiponectin-mediated neuroinflammation.

In addition to *in vitro* models, *in vivo* models also support a neuroprotective role of adiponectin against pathological markers of AD. A single intraperitoneal injection of the adiponectin receptor agonist, osmotin, improves performance in the Y-maze task in mice exposed to A*β*_1–42_ peptides [118]. In addition, the osmotin-exposed mice demonstrate attenuated expression of A*β*, beta-secretase 1, and p-tau and increased expression of the presynaptic proteins synaptophysin and synaptosomal-associated protein 25. It is interesting to note that only one dose of osmotin is needed to produce these changes in protein expression. Osmotin also reduces A*β* production in APPswe-transfected SH-SY5Y cells through a pathway dependent on AMPK [119]. Furthermore, in osmotin-treated APP/PS1 mice, A*β* expression is reduced and LTP deficits reversed in the hippocampal CA1 region. Taken together, these results indicate that adiponectin signaling is neuroprotective, especially in models of A*β* neurotoxicity.

### 4.6. Summary

In summary, adiponectin demonstrates a number of neuroprotective properties and also appears to directly influence hippocampal synapses and synaptic plasticity in a subregion-specific manner. This highlights an important role for adiponectin in modulating development of neurocognitive disorders and influencing severity. Further delineation of the role of adiponectin signaling in the brain, particularly the role of specific receptors, is needed.

## 5. Adiponectin in Anxiety and Depression

### 5.1. Overview and Adiponectin Levels in Depression

Statistics from the Anxiety and Depression Association of America state that anxiety disorders are one of the most common mental illnesses, affecting 40 million adults, or around 18% of the population, yearly in the United States. Anxiety disorders and depression often coexist, and nearly half of those diagnosed with depression are also diagnosed with an anxiety disorder. A recent meta-analysis found a significant reduction in adiponectin plasma levels in patients with depression compared to those without the disorder [120]. Although Weber-Hamann et al. reported no changes in plasma adiponectin concentrations of depressed patients during 6 weeks of antidepressant treatment [121], Narita et al. showed that plasma adiponectin levels were higher in depressed patients treated with an antidepressant drug for more than 12 months [122], suggesting time-dependent effects in antidepressant-induced alterations in adiponectin levels. However, variations such as age, BMI, additional medications, and other comorbid factors, like heart disease or diabetes, should be kept in mind while interpreting these data, as all have been demonstrated to affect circulating adiponectin levels. As evidence for a role for adiponectin in anxiety disorders, APN-KO mice demonstrate increased anxiety in an open-field test at ages 9 months and 18 months [103]. Further delineation of a specific role for adiponectin in anxiety and/or depressive disorders is needed, as well as more mechanistic studies to help in designing therapeutic strategies around adiponectin signaling.

### 5.2. Adiponectin and the HPA Axis

There are many potential mechanisms for adiponectin's role in anxiety and/or depression. One hypothesis involves adiponectin suppression of TNF*α*, a potent proinflammatory cytokine, which typically activates the hypothalamo-pituitary-adrenocortical (HPA) axis. Overactivity of HPA axis contributes to the pathogenesis of depression, in that activation of the HPA axis leads to activation of neuronal serotonin transporters leading to reduced serotonin availability in the brain [123]. TNF-*α* is thought to play an important role in the pathogenesis of depression by activating the HPA axis. Adiponectin suppresses the production TNF*α* and induces production of various anti-inflammatory cytokines [124]. Further supporting this, an inverse correlation between adiponectin and circulating TNF-*α* concentrations exists [122]. Thus, decreased production of adiponectin can lead to dysregulation of cytokines, which could contribute to depression.

Cortisol, a glucocorticoid, is released with activation of the HPA axis. Interestingly, exogenous glucocorticoid exposure appears to have an inhibitory effect on adiponectin expression [125, 126]. Therefore, in the case of pathological overactivation of the HPA axis, adiponectin expression may be reduced, leading to absence of the inhibitory effect of adiponectin on the HPA axis. However, contradictory to this, patients with higher levels of adiponectin had higher levels of cortisol [127]. In addition, adiponectin levels follow a similar diurnal variation of cortisol, which suggests that adiponectin and cortisol might be influenced by common regulatory factors [128]. Therefore, although cortisol appears to suppress adiponectin expression in case of exogenous administration and adiponectin would be expected to suppress cortisol based on effects on the HPA axis, these effects may either “cancel out” *in vivo* or only become physiologically relevant in the presence of additional factors. In summary, adiponectin appears to reduce activation of the HPA axis by reduction of TNF*α*, but the relationship between cortisol and adiponectin is less clear.

### 5.3. Adiponectin and Thyroid Hormone Regulation

Adiponectin may also exhibit a relationship with the thyroid axis. As hypothyroidism can contribute to depression and hyperthyroidism can contribute to anxiety, the effects of adiponectin on the thyroid axis are also of interest. Some conflicting data have been observed between adiponectin levels and the thyroid axis. Subjects in the highest quartile of adiponectin levels also have higher circulating concentrations of free thyroxine in serum [127]_._. However, another study showed that adiponectin was 2-fold higher in hypothyroid than in healthy patients [129]. Studies in hypothyroid dogs and guinea pigs show elevated levels of serum adiponectin, as well as their reduction after thyroid hormone replacement therapy [130]. These data indicate that both hyperthyroidism and hypothyroidism may be associated with increased adiponectin, but there is currently little known about the effects of thyroid hormones on adiponectin gene expression and vice versa.

### 5.4. Behavioral Effects of Adiponectin

While i.c.v. injection of an adiponectin neutralizing antibody in a diabetic mouse model produces stress-induced depressive- behavior, exogenous adiponectin administration via i.c.v leads to antidepressant- behavioral effects [131]. Thus, it can be hypothesized that drugs that increase endogenous adiponectin levels may also be beneficial to treat depression. Studies in a mouse model of depression showed that the effects of physical exercise in reducing depression symptoms might be mediated by adiponectin, which in turn promotes hippocampal neurogenesis [26]. Furthermore, the antidepressant activity of the PPAR*γ* agonists may be due to increased expression of adiponectin. The PPAR*γ* agonist rosiglitazone has antidepressant and anxiolytic effects, which are absent in mice lacking adiponectin [132]. In addition, a clinical trial demonstrated improvement in major depressive disorder in patients with comorbid metabolic syndrome following treatment with the PPAR*γ* agonist, pioglitazone [133].

Although the mechanisms responsible for the antidepressant effects of adiponectin are unclear, a recent animal study suggests that adiponectin signaling, specifically through AdipoR2, enhances extinction of fear memories [27]. This effect of adiponectin could be beneficial in treatment of posttraumatic stress disorder (PTSD). In this study, hippocampal infusion of adiponectin facilitated the extinction of contextual fear, but did not affect the acquisition of fear memories. Furthermore, deletion of AdipoR2, but not AdipoR1, reduced fear extinction [27]. Based on these observations, increasing adiponectin production or activating AdipoR2 receptors might be useful for facilitating extinction-based exposure treatments in managing PTSD and other trauma- and stress-related disorders.

### 5.5. Summary

At present, existing evidence concerning adiponectin and its relationship to depression and anxiety is somewhat controversial. In population-based studies, there appears to be a correlation between adiponectin levels and depression, and adiponectin may indirectly influence anxiety and depression through its actions on the HPA axis and/or thyroid-mediated effects. Additionally, adiponectin's direct effects on neurogenesis and extinction learning support continued research in this area with the goal of developing novel treatments for anxiety and depressive disorders.

## 6. Conclusion

Accumulating evidence indicates that adiponectin and adiponectin receptors may be important targets for translational studies to find novel treatments and/or prevention strategies for various CNS disorders, such as stroke-induced cerebrovascular dysfunction, AD, and anxiety and/or depressive disorders. Clearly, additional research is needed to more thoroughly examine adiponectin receptor signaling in the brain and roles of specific receptors in treatment of disease. Development of specific adiponectin receptor agonists and antagonists will be highly valuable in differentiation of these roles. We predict that adiponectin receptor signaling will be targeted in future clinical trials as a therapeutic strategy for CNS disorders.

## Figures and Tables

**Table 1:** Major animal and *in vitro* studies regarding neuroprotective effects of adiponectin in various CNS disorders.

Related pathology	Model	Treatment	Key effect(s) and mechanism(s)	Reference
Stroke	Male apoE^−/−^ mice (12–14 weeks)	Ad-APN (i.v.) 14 days prior to measurements	Effects: Ad-APN treatment reduced atherosclerotic lesion size, reduced the diameter of lipid droplets, and reduced VCAM-1 mRNA	Okamoto [66]

Stroke	Male AdipoKO, eNOS KO, and WT mice (10–12 weeks)	Ad-APN (i.v.) 5 days prior to ischemia (1 hour of middle cerebral artery occlusion followed by 23 hours of reperfusion)	Effects**:** larger infarct size in AdipoKO mice compared to WT; reduced infarct size in AdipoKO and WT mice when pretreated with Ad-APN	Nishimura [69]
			Mechanism: adiponectin reduces infarct size through eNOS; there was no alteration in infarct size in eNOS KO mice pretreated with Ad-APN	

Stroke	Male CD-1 mice (young: 3 months; age: 22–24 months)	AAV-APN (striatal injection) 7 days prior to transient middle cerebral artery occlusion	Effects: AAV-APN treatment improved motor function (neurological score, beam walk test, rotarod test, and corner test) and increased angiogenesis in young and aged mice	Miao [68]

Alzheimer's disease	SH-SY5Y_swAPP_ cell line	Adiponectin (10 *μ*g/ml) for 2 h followed by hydrogen peroxide (200–800 *μM*)	Effect: improved neuronal survival with adiponectin incubation prior to insult	Chan [115]
			Mechanisms: adiponectin is neuroprotective against oxidative stress via AdipoR1, AdipoR2, and APPL1; improved neuronal survival was prevented by knockdown of AdipoR1, AdipoR2, or APPL1	

Alzheimer's disease	Male C57BL/6J mice (8–14 weeks)	Osmotin (15 mg/kg, i.p.) at 3 or 40 days following administration of A*β*_1–42_ ( i.c.v.)	Effects: osmotin treatment improved Y-maze spontaneous alternations performance, reduced A*β*_1–42_ accumulation, and reduced tau hyperphosphorylation	Ali [118]

Alzheimer's disease	Male AdipoKO and WT mice (9 and 18 months)		Effects: AdipoKO mice had impaired performance on MWM and increased expression of A*β*_1–42_, hyperphosphorylated tau, and pIRS-1^S616^ at 18 months	Ng [103]

Alzheimer's disease	SH-SY5Y_swAPP_ cell line	Adiponectin (10 *μ*g/ml, trimeric)	Effects: adiponectin reduced A*β*_1–42_ expression and increased insulin sensitivity	Ng [103]
			Mechanisms: adiponectin reduces A*β*_1–42_ through AdipoR1 and increases insulin sensitivity through AdipoR1 and AMPK; effects blocked by knockdown of AdipoR1 or inhibition of AMPK	

Alzheimer's disease	Male APP/PS1 mice (5–12 months)	Osmotin (12 mg/kg/day i.p.) for 2 days or osmotin (5 mg/kg/day, i.p.) twice a week for 2–4 weeks	Effects: osmotin treatment increased NMDAR expression, improved hippocampal CA1 LTP, and improved performance on MWM	Shah [119]

Alzheimer's disease	SH-SY5Y_swAPP_ cell line	Osmotin (0.2 *μ*M) for 24 hours	Effects: osmotin reduced expression of A*β* and increased expression of AMPK and SIRT1	Shah [119]
			Mechanism**:** osmotin alters expression of these proteins via AdipoR1; knockdown of AdipoR1 prevented these effects	

Alzheimer's disease	Male C57BL/6J mice (5–11 weeks)	AdipoR1 knockdown (shRNA, i.v., weekly injection)	Effects: AdipoR1 knockdown mice displayed impaired performance on MWM, increased body weight, increased A*β*, and reduced pAMPK^T172^	Kim [104]

Depression	Male Adipo^+/−^ and WT mice (9–12 weeks)	Globular (0.3 *μ*g, i.c.v.) or full length (1 *μ*g, i.c.v.) adiponectin	Effects: adiponectin deficient mice displayed increased susceptibility to depressive behaviors (social interaction, sucrose preference test, and learned helplessness following a social defeat paradigm); globular or full length adiponectin had antidepressant effect in WT mice (forced swim test)	Liu [132]

Depression	Male AdipoKO and WT mice (8-9 weeks)		Effects: running enhanced hippocampal neurogenesis and increased hippocampal adiponectin in WT but not AdipoKO mice	Yau [26]

Depression	Neuro2a cell line	Trimeric adiponectin (3 *μ*g/ml)	Effect: adiponectin incubation promoted cell proliferation	Yau [26]
			Mechanism: adiponectin promotes cell proliferation through AdipoR1 in the Neuro2a cell line; siRNA knockdown of AdipoR1 but not AdipoR2 abolished adiponectin induced proliferation	

Anxiety	Male AdipoKO and WT mice (9 and 18 months)		Effect**:** increased anxiety behavior in 9- and 18-month AdipoKO mice (open-field test)	Ng [103]

PTSD	Male AdipoKO, AdipoR1 conditional KO, AdipoR2 KO, and WT mice (8–2 weeks)	Adiponectin (0.25 *μ*g, intra-DG infusion) daily 3 days prior to fear extinction and 30 min prior to each extinction session	Effects: AdipoKO and AdipoR2 KO mice, but not AdipoR1 conditional KO mice, displayed slower contextual fear extinction learning; adiponectin infusion to DG of hippocampus facilitated extinction learning in WT mice	Zhang [27]
			Mechanism: adiponectin facilitations extinction learning via AdipoR2; adiponectin infusion failed to facilitate extinction learning in AdipoR2 KO mice	

Misc.	Primary hippocampal neurons from Sprague-Dawley rats	Adiponectin (5 or 20 *μ*g/ml) for 48 h followed by kainic acid (100 *μ*M) for 12 h	Effects: adiponectin incubation improved neuronal survival, reduced expression of reactive oxygen species, and reduced caspase-3 activity	Qiu [114]
			Mechanism: adiponectin promotes neuronal survival through AMPK; adiponectin enhancement of survival was prevented by an AMPK inhibitor	

Misc.	Primary neuronal stem cells from ICR mice	Adiponectin (30 *μ*g/ml) for 4 days prior to high glucose (120 mM)	Effects: adiponectin incubation enhanced neurogenesis, increased AdipoR1 expression, and increased TLX expression	Song [28]

A*β*: amyloid beta; AAV-APN: adeno-associated virus expressing adiponectin; Ad-APN: adenovirus expressing adiponectin; AdipoKO: adiponectin knockout; AdipoR1: adiponectin receptor 1; AdipoR2: adiponectin receptor 2; AMPK: 5′ AMP-activated protein kinase; apoE: apolipoprotein E; APPL1: adaptor protein, phosphotyrosine interacting with PH domain, and leucine zipper 1; CA1: cornu ammonis 1; DG: dentate gyrus; eNOS: endothelial nitric oxide synthase; i.c.v.: intracerebroventricular; i.p.: intraperitoneal; i.v.: intravenous; IRS-1: insulin receptor substrate 1; KO: knockout; LTP: long-term potentiation; Misc.: miscellaneous; MWM: Morris water maze; NMDAR: N-methyl-D-aspartate receptor; SIRT1: sirtuin 1; TLX: tailless; VCAM-1: vascular cell adhesion molecule 1; WT: wild type.
